# The Central Role of Anti-IL-1 Blockade in the Treatment of Monogenic and Multi-Factorial Autoinflammatory Diseases

**DOI:** 10.3389/fimmu.2013.00351

**Published:** 2013-10-31

**Authors:** Silvia Federici, Alberto Martini, Marco Gattorno

**Affiliations:** ^1^2nd Division of Pediatrics, G. Gaslini Institute, Genoa, Italy; ^2^Department of Pediatrics, University of Genoa, Genoa, Italy

**Keywords:** IL-1β, autoinflammatory diseases, periodic fevers, inflammasome, treatment

## Abstract

Inherited autoinflammatory diseases are secondary to mutations of proteins playing a pivotal role in the regulation of the innate immunity leading to seemingly unprovoked episodes of inflammation. The understanding of the molecular pathways involved in these disorders has shed new lights on the pattern of activation and maintenance of the inflammatory response and disclosed new molecular therapeutic targets. Cryopyrin-associated periodic syndrome (CAPS) represents the prototype of an autoinflammatory disease. The study of the pathophysiological consequence of mutations in the cryopyrin gene (*NLRP3*) allowed the identification of intracellular pathways responsible for the activation and secretion of the potent inflammatory cytokine interleukin-1β (IL-1β). It became clear that several multi-factorial inflammatory conditions display a number of pathogenic and clinical similarities with inherited autoinflammatory diseases. The dramatic effect of interleukin-1 (IL-1) blockade in CAPS opened new perspectives for the treatment of other inherited and multi-factorial autoinflammatory disorders. Several IL-1 blockers are now available on the market. In this review we outline the more recent novelties in the treatment with different IL-1 blockers in inherited and multi-factorial autoinflammatory diseases.

## Introduction

The Autoinflammatory Syndromes are a number of different conditions characterized by episodes of inflammation secondary to an activation of the innate arm of the immune response, in the absence of high-titer auto-antibodies or antigen-specific T cells (1). The term “Autoinflammatory diseases” was originally referred to a limited number of rare inherited diseases identified as *periodic fevers*. Under this term were gathered some monogenic diseases featured by periodic or recurrent episodes of systemic inflammation causing fever often associated with rash, serositis, lymphadenopathy, arthritis, and other clinical manifestations. These disorders were secondary to mutations of genes coding for proteins that play a pivotal role in the regulation of the inflammatory response. In the following years the discovery of new genes and novel inherited conditions allowed to clarify that the possible clinical presentation of these new diseases was much wider and that the term of periodic fevers was associated to a reductivist view of the clinical phenotype possibly associated to this group of disorders (Table 1).

**Table 1:** **Clinical classification of inherited and multi-factorial autoinflammatory diseases (AID)**.

	Inherited AID (gene, transmission)	Multi-factorial AID
**CLINICAL PRESENTATION**		
Recurrent episodes of inflammation	FMF (*MEFV*, AR) TRAPS (*TNFRSF1A*, AD) MVK (*MVK*, AR)	PFAPA
		Recurrent idiopathic pericarditis
		Mollaret syndrome (recurrent meningitis)
Systemic inflammation with urticarial rash	CINCA/NOMID (*NLRP3*, AD) Muckle–Wells/FCAS (*NLRP3*, AD) FCAS2 (*NLRP12*, AD)	SoJIA
		Adult onset Still disease
		Schnitzler’s syndrome
		Delayed pressure urticaria
Sterile inflammation of skin/bone/joints	PAPA (*CD2BP1*, AD)	CRMO
	DIRA (*IL1RN*, AR)	SAPHO
	DITRA (*IL36RN*, AR)	Gout and pseudogout
	Majeed syndrome (*LPIN2*, AR)	HLA-B27 spondyloarthropathy
	CAMPS (*CARD14*, AD)	Reactive arthritis
	Blau’s syndrome (*CARD15*, AD)	Sweet syndrome
		Generalized pustular psoriasis
		Hallopeau acrodermatitis
Panniculitis/lipodystrophy	Nakajo–Nishimura (*PSMB8*, AR)	Neutrophilic panniculitis
	JMP (*PSMB8*, AR)	Erythema nodosum and panniculitis
	CANDLE syndrome (*PSMB8*, AR)	
Inflammatory bowel disease	Early-onset inflammatory bowel disease (*IL10*, *IL10RA*, *IL10RB*)	Crohn’s disease
Hemophagocytic lymphohistiocytosis	FHL1 (Unknown)	SoJIA-associated MAS
	FHL2 (*PFR1*/perforin 1, AR)	Infection-associated MAS
	FHL3 (*UNC13D/Munc* 13-4, AR)
	FHL4 (*STX11*/syntaxin 11, AR)
	FHL5 (*STXB2*/syntaxin binding protein, AR)

FMF, familial Mediterranean fever; FCAS, familiar cold autoinflammatory syndrome; MWS, Muckle–Wells syndrome; TRAPS, TNF-receptos associated periodic syndrome; MVK, mevalonate kinase deficiency; CINCA, chronic infantile neurological cutaneous and articular; PAPA, pyogenic sterile arthritis, pyoderma gangrenosum, and acne; JMP, joint contractures, muscle atrophy, microcytic anemia, and panniculitis-induced childhood-onset lipodystrophy; CANDLE, chronic atypical neutrophilic dermatosis with lipodystrophy and elevated temperature; DIRA, deficiency of the IL-1 receptor antagonist; DITRA, deficiency of IL-36 receptor antagonist; CAMPS, CARD14-mediated pustular psoriasis; FHL, familial hemophagocytic lymphohistiocytosis; MAS, macrophage activation syndrome; AR, autosomal recessive; AD, autosomal dominant.

In the meanwhile, the pathogenetic insights derived from studies on these rare disorders allowed a better understanding of mechanisms responsible for the induction and maintenance of inflammation and have set the basis for the identification of molecular targets for treatment. Due to the enormous interest raised by the identification of *NLRP3* Inflammasome (2) and by the dramatic response to interleukin-1 (IL-1) blockers observed in patients presenting a gain of function mutation of *NLRP3* gene (cryopyrin-associated periodic syndromes, CAPSs) (3–5), IL-1, is now considered the pivotal pro-inflammatory cytokine in these disorders. The availability of specific IL-1 targeting agents has revealed a pathological role of IL-1-mediated inflammation in a growing list of multi-factorial diseases in which a deregulation of the same intracellular pathway probably occurs. Aim of the present review is to provide a state of the art of the treatment of inherited and multi-factorial autoinflammatory diseases with IL-1 blockers.

## Anti-IL-1 Agents

**Figure 1:**
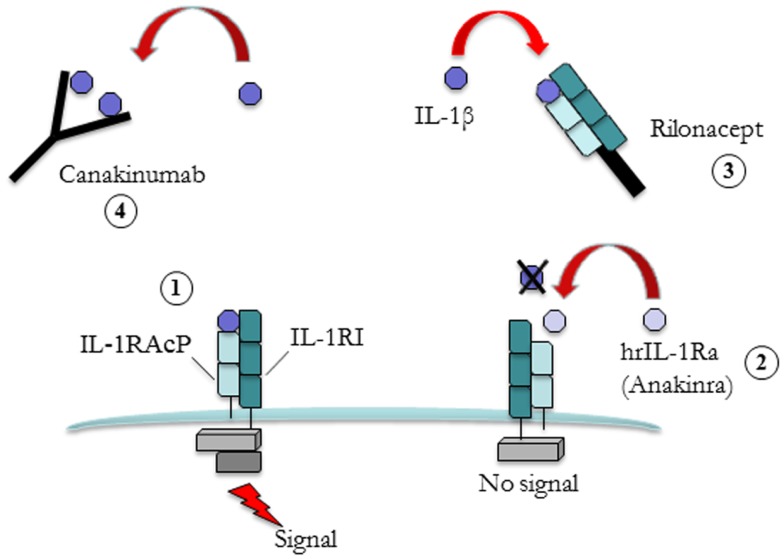
**Different strategies for IL-1 blockade. Free interleukin (IL)-1b binds to type 1 IL-1 receptor (IL-1R1) and to the adaptor protein IL-1RAcP leads to signal transduction (1)**. Human recombinant IL-1 receptor antagonist (hrIL-1Ra, Anakinra) (2) competes with free IL-1β for the binding with IL-1R1 but not with the adaptor protein, thus preventing signal transduction. Rilonacept (3) is a fusion protein comprising the extracellular domains of the IL-1β receptor (IL-1RI) and adaptor protein (IL-1RAcP) attached to a human IgG molecule. Its action is to bind to circulating IL-1β. The same mechanism of action is also valid for Canakinumab (4), a fully humanized anti-IL-1 monoclonal antibody.

There are two related but distinct IL-1 genes, *IL1A* and *IL1B*, encoding IL-1α and IL-1β, respectively. The IL-1α precursor is constitutively present, in an active form, in most of the cells of healthy individuals. Under disease conditions, IL-1α moves to the cell’s surface membrane where it can activate adjacent cells bearing the IL-1 receptor (6, 7). Conversely IL-1β is a product of a limited type of cells (e.g., blood monocytes, tissue macrophages, and dendritic cells). In physiological conditions IL-1β is in an inactive form and requires a series of intracellular events to be activated. In normal conditions both IL-1α and IL-1β bind to type 1 IL-1 receptor (IL-1R1) and to the adaptor protein IL-1RAcP in order to trigger signal transduction (Figure 1).

Targeting IL-1 started in 1993 with the introduction of a recombinant non-glycosylated form of the naturally occurring IL-1 receptor antagonist (IL-1Ra, Anakinra), which blocks the activity of both IL-1α and IL-1β. IL-1Ra competes with free IL-1α and IL-1β for the IL-1R1 binding but not with the adaptor protein, thus preventing signal transduction. Anakinra has a short terminal half-life ranging from 4 to 6 h and it is administered subcutaneously daily. Other IL-1 blockers have been successively developed. Rilonacept is a protein consisting of the extracellular domains of humanized IL-1 type 1 receptor and the IL-1 receptor accessory protein fused with the Fc portion of IgG1. Rilonacept which has a terminal half-life of 6.3–8.6 days and is administered once weekly, binds IL-1β and IL-1α with high affinity and powerfully inhibits IL-1 activity.

Canakinumab is a fully human anti-interleukin-1β (IL-1β) monoclonal antibody that selectively blocks IL-1β with high affinity and does not cross-react with IL-1α or IL-1Ra. Binding to IL-1β prevents the cytokine from the interaction with its receptor and, thus, blocks the inflammatory signaling cascade. Compared to other IL-1 blockers, Canakinumab provides a longer plasma half-life (21–28 days).

Recently a novel compound called Gevokizumab has been developed. Gevokizumab is a IgG2 humanized mAb that modulates IL-1β bioactivity by reducing the affinity for its IL-1RI:IL-1RAcP signaling complex. It binds to a single IL-1β epitope where residues critical for binding have been identified. It has a long plasma half-life, which would allow once-monthly administration. Some clinical trials are ongoing in osteoarthritis, non-infectious uveitis, Pyoderma Gangrenosum, and Diabetes mellitus^1^.

Other therapeutic approaches, including IL-1α neutralization, a therapeutic vaccine targeting IL-1β, and a chimeric IL-1Ra, are in early clinical trials. Moreover, orally active small-molecule inhibitors of IL-1 production, such as Caspase 1 inhibitors, have been developed and are being tested (8, 9).

## Monogenic Autoinflammatory Diseases

### Cryopyrin-associated periodic syndrome

Familiar Cold Autoinflammatory Syndrome (FCAS), Muckle–Wells Syndrome (MWS), and Chronic Infantile Neurological Cutaneous and Articular Syndrome (CINCA) or Neonatal Onset Multi-systemic Inflammatory Disease (NOMID) are three diseases originally described as distinct entities that turned out to belong to the wide clinical phenotype of disorders due to mutations of *NLRP3* gene (NOD-like receptor 3, previously known as Cold-Induced Autoinflammatory Syndrome 1, *CIAS1*). Mutations in this site determine a gain of function of the protein with subsequent increased secretion of IL-1β. FCAS represents the milder phenotype and presents with cold-induced fever, urticaria-like rash, and constitutional symptoms. MWS represent an intermediate form and manifests with fever, urticarial rash, sensorineural hearing loss, and arthritis usually not related to cold exposure. CINCA patients, instead, display the worst clinical picture characterized by fever, urticarial rash, epiphyseal overgrowth of the long bones, and central nervous system involvement (mental retardation, chronic aseptic meningitis, increased intracranial pressure, papilledema, cerebral atrophy, sensorineural hearing loss). Almost 1/3 of patients with CAPS may develop amyloidosis that seem to be more frequent in MWS than in FCAS or CINCA patients.

The active form of cryopyrin (NLRP3) is involved in the assembly of an intracellular multi-protein complex (called inflammasome) that play a pivotal role in the activation of Caspase 1, a cytoplasmatic enzyme responsible for the activation and secretion of the biological active 17 kD form of IL-1β (10).

The massive secretion of active IL-1β observed in cryopyrin-mutated individuals, suggested that anti-IL-1 treatment could represent an effective therapy (10).

Initial isolated case reports showed the dramatic effects of Anakinra in the control of rash and other systemic manifestations in MWS (3), FCAS (4), and CINCA (5, 11, 12) patients.

The long-term efficacy and safety of Anakinra in pediatric CAPS patients has been described in two distinct cohorts of patients (13, 14).

These two studies indicate that Anakinra treatment is safe and effective in the long term and should be initiated early, before irreversible lesions have developed.

Sibley et al. recently published an open label, long-term follow-up study on a cohort of 26 CINCA/NOMID patients treated with Anakinra 1–5 mg/kg/day for at least 36 months (15). Twenty-one out of 26 patients carried mutations in *NLRP3* gene and all of them had an active disease at baseline.

Aim of the study was to evaluate the efficacy and safety of 36 and 60 months of IL-1-blocking therapy in controlling systemic and organ-specific inflammation and in preventing the progression of organ damage.

Sustained improvements in diary scores, parent’s/patient’s and physician’s global scores of disease activity, parent’s/patient’s pain scores, and inflammatory markers were observed during all the period of the study. Despite a general good control of clinical manifestations (including hearing loss, ocular manifestations, and headache) and laboratory parameters, a few patients displayed a persistent even if mild inflammation of CNS. Anti-IL-1 treatment did not prevent the progression of the bone involvement. Overall Anakinra was well tolerated and no major adverse effects were observed.

Kuemmerle-Deschner et al. reported the long-term safety and efficacy of Anakinra in pediatric and adult patients affected by MWS. A rapid and persistent control of constitutional symptoms and organ manifestations was observed (16).

The efficacy of Rilonacept (160 mg/weekly) on CAPS-related clinical manifestations have been shown in two sequential placebo-controlled studies performed in patients with FCAS or MWS (17). The treatment was generally well tolerated. Site reactions in 1/3 of patients and mild upper respiratory infections were the most common adverse events (AEs). In 2008, the FDA in USA has approved its use in CAPS as an orphan drug in adult and children above the age of 12.

The first evidence for the efficacy of Canakinumab was obtained by a 48-week, double-blind, placebo-controlled, randomized withdrawal study, involving 35 MWS patients receiving a subcutaneous dose of 150 mg (or 2 mg/kg) every 8 weeks (18).

These positive results were confirmed in a 2-years open label study in which the response to treatment was analyzed in 166 patients (109 Canakinumab-naive and 57 roll-over patients) with FCAS (*n* = 30), MWS (*n* = 103), or NOMID/CINCA (*n* = 32) (19). The study showed that a complete response was achieved in 85 of 109 Canakinumab-naive patients (78%; 79/85 patients within 8 days, and 5 patients between days 10 and 21). Of 141 patients with an available relapse assessment, 90% did not relapse, their CRP/SAA levels normalized ( <10 mg/l) by day 8, and remained in the normal range thereafter. Median treatment duration was 414 days (29–687 days). Notably, upward adjustments of dose or frequency were needed in 24.1% patients mostly children with a severe CAPS phenotype (CINCA/NOMID). Predominant AEs were infections (65.7%) of mostly mild-to-moderate severity. Serious AE reported in 18 patients (10.8%) were mainly infections and were responsive to standard treatment. The majority of patients (92%) reported as having no injection-site reactions and only 8% of patients reported mild-to-moderate reactions. Patients receiving vaccinations (15%) showed normal immune response. Based on these studies, Canakinumab has been approved in many countries for all forms of CAPS in patients older than 4 years. The first experience on the use of Canakinumab in daily clinical practice in 13 pediatric CAPS patients has been recently reported (20). Globally, patients with a mild-intermediate MWS phenotype display a complete control of disease activity maintaining the initial dosage of 2 mg/kg (or 150 mg) every 8 weeks, independently from their age. Conversely, the majority of CINCA patients required to increase the dosage up to 4 mg/kg (or 300 mg) and progressively increase the frequency of dosing (20).

### Familial Mediterranean fever

Familial Mediterranean fever (FMF) is the most frequent among hereditary recurrent inflammatory disorders. It presents with an autosomal recessive pattern of inheritance and is due to mutations in the *MEFV* (Mediterranean Fever) gene encoding pyrin (also called marenostrin) (21, 22).

Colchicine represents the treatment of choice for FMF (23). Nonetheless, approximately one third of the patients have a partial remission and about 5–10% are non-responders; another 2–5% do not tolerate the drug mainly due to gastrointestinal symptoms (24). Data from a large international registry (Eurofever) showed that almost 40% of FMF patients display an incomplete response to colchicine, by means of persistent presence of fever attacks or persistent elevation of acute-phase reactants (25).

Before the advent of colchicine, reactive AA amyloidosis represented the most frequent and severe complication of the disease. It occurred in almost 60–75% of patients over the age of 40 with a poor prognosis. Amyloidosis usually presents in those patients with severe attacks starting early in infancy but it may develop even in those patients without clear inflammatory episodes. The genetic background, the presence of high penetrance mutations, environmental factors, and the presence of SAA1 gene haplotype seem to influence the development of amyloidosis too. Even if the use of Colchicine dramatically reduced the incidence of amyloidosis, a relevant number of patients still present this long-term complication (26).

Recent evidences have shown that pyrin is able to interact with some components of the *NLRP3* Inflammasome (e.g., ASC and Caspase 1), raising the hypothesis of a possible role of this protein as a negative regulator (27, 28) or as an inducer of IL-1β secretion (29–31). Omenetti et al. have recently reported that *MEFV*-mutated monocytes display an increased IL-1β secretion (32).This over-secretion is correlated to the number and penetrance of *MEFV* mutations (32), confirming the presence of a dose effect of *MEFV* mutations already suggested by studies on FMF animal models (29) and patients (33).

Indeed, the use of IL-1 targeting drugs in colchicine-resistant FMF patients was recently proposed as a valid therapeutic strategy (27, 34). Anakinra and Canakinumab, have been reported to be generally effective in case reports and non-controlled series in more than 30 patients with colchicine-resistant or intolerant FMF (35–44).

Blocking IL-1 has been used with good results even in a small cohort of patients with AA amyloidosis and chronic renal failure (44). In the majority of cases Colchicine was maintained after the introduction of Anakinra even if at a lower dose.

Normally SAA is completely degraded in the lysosome. It seems that, in patients with AA amyloidosis, the process of degradation may be impaired. High levels of SAA due to inflammatory burden thus worsen the accumulation of this substance. A good control of the disease by IL-1 blockers with persistent SAA value in the range of normality, may avoid the worsening of amyloidosis and may allowed the progressive degradation of the fibrils previously accumulated.

Recently, Hashkes et al. reported the results of a randomized, double-blind, single-participant alternating treatment study for Rilonacept in 14 colchicine-resistant or intolerant FMF patients (45). Participants were aged 4 years or older and were required to have an estimated mean of 1 or more FMF attacks per month for 3 months before screening and 1 or more attacks per month during screening despite receiving adequate colchicine treatment. Colchicine intolerance was defined as the inability to tolerate the drug at that dose controlling attacks to fewer than 1 per month. Patients were treated with Rilonacept 2.2 mg/kg (maximum, 160 mg) or equal volume of placebo, both given once weekly by subcutaneous injection. Rilonacept significantly reduced the frequency of FMF attacks versus placebo and ameliorated the physical Health related quality of life.

### TNF-receptor associated autoinflammatory syndrome

TNF-receptor associated autoinflammatory syndrome (TRAPS) is a rare dominantly inherited disorder, caused by mutations in the p55 TNF Receptor (or TNFR1), encoded by the TNF Receptor Super Family 1A (*TNFRSF1A*) gene (46). Fever episodes are usually prolonged (1–3 weeks) and are usually accompanied by serositis, arthritis, a skin rash with underlying fasciitis, and periorbital edema. Reactive amyloidosis develops in almost 14–25% of TRAPS patients and it seems to correlate with the severity and lasting of fever episodes.

Mutations of TNFR1 lead to a misfolding of the protein that is thus accumulated in the ER and cytoplasm. This leads to a response to the unfolded protein with consequent inappropriate cytokine secretion (47). Mitochondrial reactive oxygen species promote production of pro-inflammatory cytokines and are elevated in TNFR1-TRAPS (48). Moreover, TRAPS patients display an exhaustion of the autophagy system due to the intracellular overload of the unfolded mutated protein that represents a further “stress signal” for the cells (49). This may lead to the activation of the NLRP3 Inflammasome, consistent with the over-secretion of active IL-1β observed in TRAPS patients (49).

The inflammatory attacks are usually responsive to high-dose corticosteroids but side effects limit their use especially in patients with frequent relapses or nearly continuous symptoms (chronic course). The use of immunosuppressive drugs have been reported to be ineffective (50). Since the molecular defect of p55 TNFR is also associated with an impaired shedding of the receptor from the membrane surface, the use of Etanercept (Enbrel), was originally proposed (46).

In a recent study Bulua et al. (51) reported the experience of 15 TRAPS patients enrolled in a prospective, open label, dose-escalation study using Etanercept. The treatment significantly attenuated the total symptom score, as well as reduced the frequency of symptoms and the values of acute-phase reactants during asymptomatic periods. However, during a 10-year follow-up period, most of the patients discontinued treatment mainly due to the lack of efficacy, with a median duration of treatment of 3.3 years.

Of note, the use of anti-TNF monoclonal antibodies (infliximab and adalimumab) has been shown to worsen the inflammatory manifestations in TRAPS patients (52–54).

On the other hand, some anecdotic observations have suggested an excellent response to Anakinra in some patients (53, 55).

In 2008, the first small cohort of TRAPS patients treated with Anakinra was described (56). Patients were treated with Anakinra 1.5 mg/kg/day. All patients had a dramatic response, with disappearance of symptoms and normalization of acute-phase reactants. During the following year the patients were treated continuously with Anakinra and did not experience any disease-related clinical manifestations or any increase in acute-phase reactants (56).

Interestingly, data from the Eurofever registry recently showed the better performance of IL-1 blockade on anti-TNF treatment in TRAPS patients. In fact, even if Etanercept was beneficial in 32 of the 37 patients, only 11 (30%) experienced a complete response. Conversely, Anakinra was able to induce a complete response (absence of clinical manifestations and normalization of acute-phase reactants) in 26 of 33 patients (79%) and a partial response in five others (25). The effect of Anakinra in patients carrying low-penetrance mutations (e.g., R92Q) seems less striking in respect to those observed in patients with mutations affecting cysteine residues (57). The same good results have been preliminary reported in one TRAPS patients treated with the anti-IL-1 monoclonal antibody (Canakinumab) (58).

Interim data of an open label 4-months study with Canakinumab and of 5-months of follow-up involving 20 active TRAPS patients have been recently presented, showing the complete control of clinical manifestations and persistent normalization of acute-phase reactants (59).

These data support the pivotal role of IL-1β in the pathogenesis of TRAPS, but need to be confirmed in a larger number of patients.

### Mevalonate-kinase deficiency

Mevalonate-kinase deficiency (MKD) is due to mutations in the mevalonate-kinase (MVK) gene (60, 61). MVK is an essential enzyme in the isoprenoid biosynthesis pathway which produces several molecules involved in different cellular processes (62). The severe reduction of the enzymatic activity leads to a severe multi-systemic disease, named mevalonic aciduria. The partial enzymatic defect is associated with a normal mental and physical development, but is characterized by recurrent fever attacks.

Almost 25% of MKD patients present more than 12 attacks per years in their second decade of life (17.8% of patients >20 year old) with a severe impact on quality of life in some patients (63). Unlike what happen in the other monogenic periodic fevers, amyloidosis do not represent a frequent complication of MKD. The first case of AA amyloidosis was reported in 2004 (64) and few other patients in the following years (65). van der Hilst et al. (63) reported a frequency of 2.9% of amyloidosis in a group of 103 patients coming from The International hyper-IgD syndrome (HIDS) database.

Fever attacks usually respond to the administration of a single or a few steroids doses. However, due to the high frequency of fever episodes, some patients may need almost continuous treatment.

Colchicine, cyclosporine, thalidomide, and statins are not effective (63). The efficacy of biologic treatments is largely anecdotal and still controversial. Anti-TNF therapy (Etanercept) has been found to reduce the frequency and intensity of fever attacks in some patients (66–68) but not in others (69). In the same line a positive response was also observed after the use of Anakinra (70–72). The same variable response was also observed in the International HIDS registry (63).

Galeotti et al. reported the results of a retrospective study aimed at evaluating the effects on disease activity of an anti-IL-1 treatment in a group of 11 genetically confirmed MKD patients (73). In this study daily Anakinra (nine patients) or Canakinumab injections every 4–8 weeks (six patients, in four cases following Anakinra treatment) were associated with complete remission in four cases and partial remission in seven.

The rationale for the use of IL-1 blockers is found in studies from a Dutch group (74) in which the Authors show that a shortage of isoprenoid end products due to the defective function of mevalonate kinase contributes to an increased secretion of IL-1β by MK-deficient peripheral blood mononuclear cells.

In a recent paper Ter Haar et al. analyzed MKD response to treatment in 67 patients coming from The Eurofever International Registry^2^ (25). Anakinra was effective in 24 (89%) of 27 patients treated, inducing a complete remission in six (22%) of them. Etanercept was effective in 11 (65%) of 17 treated patients, with only one complete response.

An Open label, Multicenter, Pilot Study of 6-month Canakinumab Treatment With up to 6-month Follow-up in Patients With active HIDS is now ongoing to evaluate the efficacy, the safety, and the pharmacokinetics (PK)/pharmacodynamics (PD) of Canakinumab treatment in patients with HIDS (see text footnote 1).

### Blau’s syndrome or NOD2 gene-associated pediatric granulomatous arthritis

Blau’s syndrome is the genetic form of what was previously known as early-onset sarcoidosis and is due to mutations of the NACHT domain of the gene *CARD15* (or *NOD2*). It is characterized clinically by the triad of arthritis, skin rash, and uveitis, and histologically by the presence of non-caseating epithelioid granulomas in the affected sites that represent the hallmark of the disease. Other, less frequent clinical symptoms, such as camptodactyly, intermittent fever, cranial neuropathies, and malignant hypertension, have also been reported (75). The clinical course is variable, but in many cases the prognosis is poor, with severe disabilities and sequelae in a high percentage of patients. Eye involvement is frequently progressive and can lead to panuveitis and severe complications up to blindness.

An international registry was established in 2005 to collect both patients affected by Blau syndrome and its sporadic counterpart of early-onset sarcoidosis that share with the former an identical phenotype. Rose et al. (76) reported the results of the registry 1 year after its creation. In the paper the authors aimed to define the spectrum of the clinical phenotype and establish the mutation frequency and variants in patients with “pediatric granulomatous arthritis.” Up to date this work represent the largest collection of patients (33 pts) with both sporadic or familiar pedigrees.

Anti-TNF monoclonal antibodies have been shown to be effective in some anecdotal reports (77, 78). Martin et al. did not observe evidence for increased IL-1β production in cells obtained from five subjects with Blau syndrome compared with healthy control (79). In their study the Authors treat two cases with recombinant human IL-1 receptor antagonist without an evident clinical response. On the other hand few case reports have shown the effectiveness of IL-1 blockade (75, 80).

### Pyogenic sterile arthritis, pyoderma gangrenosum, and acne syndrome

Pyoderma gangrenosum and acne (PAPA) syndrome is a rare autosomal dominant inherited autoinflammatory syndrome characterized by pyogenic sterile arthritis (usually occurring after minor trauma or even spontaneously) less frequently accompanied by PAPA. It is associated with dominant missense mutations in the proline-serine-threonine phosphatase-interacting protein 1 (*PSTPIP1*) gene (81, 82). Around 30 cases have been described in the literature so far. PAPA syndrome is generally responsive to oral or intra-articular glucocorticoids (81, 82) but anti-TNF has been reported to be effective in a few patients too (83, 84).

Interestingly, *PSTPIP1* is able to interact with pyrin, the protein mutated in FMF (85). It has also been proposed that pyrin and *PSPTPIP1* form a tri-molecular complex with ASC that is able to directly recruit and activate Caspase 1 (31).

Mutated *PSTPIP1* has increased interaction with pyrin and the effect of this interaction is similar to that of mutated pyrin, resulting in decreased apoptosis and elevated IL-1 levels (85) thus setting the basis for the use of IL-1 blockade in PAPA patients.

Up to date anecdotal cases of treatment with Anakinra (both as maintenance therapy or at occurrence) has been reported with good results particularly on articular manifestations (86–88). More uncertain is the effectiveness of Anakinra on cutaneous manifestations.

Geusau et al. have recently reported the successful treatment of a patient with a PAPA-like syndrome with Canakinumab (89). This patient, despite the unusual presence of an homozygous substitution in exon 11 (c.773G > C p.Gly258Ala) in a disease with an autosomal recessive pattern of inheritance, had a clinical phenotype clearly consistent with a PAPA syndrome. He was mainly affected by cutaneous manifestations (PAPA fulminans) since the age of 14 and, despite the absence of arthritis he had suffered from early childhood of episodes of arthralgia, painful joints, and fever not responding to antibiotics. Moreover he had elevated inflammatory markers and white blood cell count with relative neutrophilia. Notably, the substitution found in this case had been previously described in an heterozygous symptomatic PAPA patient^3^. Soon after the first injection of Canakinumab the patient displayed a complete remission of the acne and a complete normalization of acute-phase reactant. Canakinumab was administered every 9 weeks and no flares were observed in a 9-months period apart from slight flares of acne at the end of 8 weeks, occasionally accompanied by a moderate increase in C-reactive protein and serum amyloid A levels (89).

In line with this experience a Phase II Multi Center Open Label Pilot Study to assess a potential effect of Canakinumab on Pyoderma Gangrenosum is ongoing (see text footnote 1).

### Deficiency of the interleukin-1-receptor antagonist

Deficiency of the interleukin-1-receptor antagonist (DIRA) is a recently identified autoinflammatory disorder characterized by a severe systemic inflammation beginning at birth with persistent inflammation and papular pustulosis. A severe inflammatory bone involvement (multifocal osteomyelitis, periostitis) is also observed. The patients display deleterious truncating or missense mutations in the interleukin-1-receptor antagonist (*IL1RN*) gene (90). Deletions at chromosome 2q13, which encompasses several IL-1 family members, including *IL1RN* have been reported too (91). As a result of these mutations, no interleukin-1-receptor antagonist protein is secreted, with subsequent unopposed action of IL-1. The patients show a dramatic response to the substitutive treatment with recombinant IL-1 receptor antagonist (Anakinra) (90). Up to date only 16 cases have been described in literature.

### Majeed’s syndrome

The Majeed’s syndrome is an autosomal recessive, autoinflammatory disorder characterized by the triad of chronic recurrent multifocal osteomyelitis (CRMO), congenital dyserythropoietic anemia, and inflammatory dermatosis. The disease was firstly described in three related Arab children by Majeed and co-workers (92) and subsequently associated to mutation of the *LPIN2* gene (93). A recent report describe the over production of IL-1 by monocytes of two brothers carrying *LPIN2* mutations and the good response to anti-IL-1 treatment (94), thus showing the possible pivotal involvement of this cytokine also in this condition.

## Multi-Factorial Disorders

The identification of genetically determined inflammatory diseases characterized by a predominant involvement of innate immunity in respect to the adaptive branch of the immune response (lack of pathogenic auto-antibodies and antigen-specific T cells) represented a major breakthrough in the field of inflammatory diseases in the last decade. It became clear that several multi-factorial inflammatory conditions were much more similar to the recently identified inherited autoinflammatory diseases rather than to the classical autoimmune rheumatic diseases (RA, SLE, etc.). Indeed, many multi-factorial disorders present with the same clinical manifestations observed in inherited autoinflammatory diseases (Table 1) and also share the same pathways of activation of innate immunity.

In fact, it was observed that NLRP3 Inflammasome can be activated not only by rare, gain of function mutations in the *NLRP3* gene but also by a large variety of endogenous or exogenous stimuli leading to an over-activation of cells of innate immunity in different circumstances (95–97). These findings represented the rationale for the successful use of the anti-IL-1 blockers in many multi-factorial inflammatory diseases.

A clear example is given by *systemic onset juvenile idiopathic arthritis* (*SoJIA*) and *adult onset Still’s disease (AOSD)* that share with the severe form of CAPS (CINCA) the multi-systemic inflammatory involvement (Table 1). SoJIA and AOSD patients classically present with high spiking fever, arthritis (either with oligoarticular or severe symmetric polyarticular course), and evanescent rash. Lymphadenopathy, hepatosplenomegaly, serositis, arthralgias, and myalgias are frequently present too. Few patients with SoJIA or AOSD respond to NSAID monotherapy. A single steroid course can control disease manifestations in patients with monocyclic presentation. However the majority of the patients present a progression of the disease and develop a steroid dependency. In these patients Methotrexate or TNF-α neutralizing agents have been employed with limited success (98, 99). IL-6 has been proven to play a pivotal role in the pathogenesis of the disease (100) and IL-6 blockade represent a very effective treatment in steroid-dependent patients, as recently confirmed by a large international multicentre trial (101). On the other hand, seminal studies by Pascual et al. were able to demonstrate the presence of a clear IL-1 signature in SoJIA patients (102, 103) that, at least in a variable percentage, display an optimal response to IL-1 blockade (98, 103–105). The same observations were also anecdotally reported in AOSD patients (106, 107). A recent large international trial has shown that the use of Canakinumab is able to control the articular and systemic manifestations and allow the tapering and withdrawn of steroids in a large proportion of patients (108).

Recurrent bouts of seemly unprovoked inflammation are the most classical manifestation observed in inherited periodic fevers (Table 1).

*PFAPA syndrome* is the prototype of a multi-factorial periodic fever syndrome. Patients display recurrent, often clock-wised, spontaneous episodes of fever in the absence of proof of infections, associated with an increase of the principal acute-phase reactant lasting 4–6 days. Usually the disease onset is before the age of five and aphthous stomatitis, pharyngitis, and laterocervical lymphadenopathy represent the most characteristic symptoms. Children present normal growth and complete well-being among the episodes. The disease is usually self-remitting with the age. The use of steroid on demand and the tonsillectomy in persistent cases represent the two main therapeutic strategies. The response to Anakinra on demand represents the *in vivo* demonstration of the *in vitro* studies indicating the involvement of this cytokine in the pathogenesis of the disease (109, 110).

*Idiopathic recurrent pericarditis* also has many features that are consistent with an autoinflammatory disease. The first observation of the role of IL-1 blockade in this condition came from the report on the effect of Anakinra in three steroid-dependent and colchicine-resistant children (111). Pericarditis recurred when Anakinra treatment was discontinued and no further episodes occurred after it was resumed. After this report several others confirmed the good response to IL-1 blockade (112–114).

Urticarial rash associated with signs of systemic inflammation represents the classical hallmark of the milder form of CAPS. *Schnitzler’s syndrome* share many clinical similarities with these conditions (Table 1). It is a chronic disabling inflammatory disorder, characterized by chronic urticarial rash, paraproteinemia, and systemic inflammation. Disease onset is usually observed after the age of 40 and patients can also present fever, bone pain, and arthralgias or arthritis. A higher risk of developing a lymphoproliferative disorder and AA amyloidosis in the long term has also been reported (115, 116). Anakinra was found to be effective in over 45 cases to date (117, 118) implying a pivotal pathophysiological role of IL-1. Canakinumab has been tried with optimal response too. A 9-month open label, single-arm trial demonstrate the long-term efficacy of Canakinumab in a cohort of 8 Dutch patients (119).

A prevalent neutrophilic inflammation of joints, bone, and skin is a common finding in a number of inherited autoinflammatory diseases (such as PAPA, DIRA, DITRA, etc.), but also in many multi-factorial disorders (Table 1).

A severe and painful arthritis characterized by a diffuse neutrophilic joint effusion is the main clinical feature of *gout and pseudogout*, two common conditions occurring in adulthood. The diseases are caused respectively by the deposition of monosodium urate (MSU) and calcium pyrophosphate dihydrate (CPPD) crystals in the joints and periarticular tissues. The finding that both MSU and CPPD crystal are able to activate the NLRP3 inflammasome (96) strongly supported the hypothesis that the inflammatory manifestations of these metabolic conditions recognize the same pathogenic mechanisms observed in inherited autoinflammatory diseases, by means of a persistent over-activation of the NLRP3 inflammasome.

IL-1 blockade has been shown to be effective in colchicine-resistant gout and pseudogout (120, 121).

Ghosh et al. described the results of Anakinra treatment in 26 patients affected from gout (122). In 67% of them, pain improved significantly within 24 h and a complete resolution of signs and symptoms of gout occurred by day 5 in 72.5% of patients. Anakinra was well tolerated and no adverse outcomes were attributed to the drug. Only one patient appeared to be refractory to this form of IL-1 inhibition (122).

In two recent 12-week randomized, multicentre, double-blind, parallel-group core studies, Canakinumab provided significant pain and inflammation relief and reduced the risk of new flares in patients with acute gouty arthritis. AEs reported more frequently with Canakinumab included infections, low neutrophil count, and low platelet count (123).

*Chronic recurrent multifocal osteomyelitis* and *Synovitis, Acne, Pustulosis, Hyperostosis, and Osteitis (SAPHO) syndrome* display a number of clinical similarities with inherited autoinflammatory diseases characterized by the presence of sterile osteolytic lesions, such as Majeed’s syndrome and DIRA.

No uniformly effective therapeutic strategies have been established for both CRMO and SAPHO (124, 125). Among the various possible biologic treatments used so far, also IL-1 blockers have been anecdotally reported to be at least partially effective in both conditions (124, 126, 127).

Pustular psoriasis and neutrophilic dermatoses are also frequent manifestations observed in inherited autoinflammatory disease, such as DIRA, DITRA, and CARD14-mediated familial psoriasis.

*Generalized pustular psoriasis* is an acute form of psoriasis with erythematous, painful skin, and widespread sterile pustules associated with systemic inflammation (fever, malaise), leukocytosis, and elevation of acute-phase reactants. The effect of IL-1 treatment have been recently described in two patients (128).

*Sweet’s syndrome* is a neutrophilic dermatosis characterized by fever, an elevated neutrophil count, and painful erythematous cutaneous lesions. Histopathological analysis reveals a neutrophilic dermal infiltrate. Systemic corticosteroid therapy remains the mainstay of the treatment. However recently have been described few cases with a dramatic response to IL-1 blockage in patients resistant to standard treatments (129, 130).

*Acrodermatitis continua of Hallopeau* (ACH) is a rare, chronic disease characterized by acropustular eruptions predominantly involving the distal phalanges of the hands and feet with marked involvement of the nail bed. The sterile pustules may coalesce to form groups of lesions, which, over time, may spread proximally to involve the dorsal side of the hands, forearms, and feet. Onychodystrophy and even anonychia of the involved digits, atrophic skin changes, and osteolysis are often present causing painful and disabling lesions. In the last years cases responsive to either anti-TNF (131) and anti-IL-1 (Anakinra) treatments (132) have been described.

In conclusion, the recent advances in the identification of the molecular mechanisms leading to the severe inflammatory response observed in ultra-rare inherited autoinflammatory diseases allow to clarify that similar pathogenic mechanisms play also a crucial role in sustaining inflammation in a growing number of multi-factorial disorders. These findings led to a relevant re-thinking in the classification of the inflammatory diseases (133) and pointed out the pivotal role of IL-1 as therapeutic target in these conditions (134). The underlined importance of IL-1 in the pathogenesis of most of these conditions is reflected by the high number of clinical trials ongoing with IL-1 blockers (see text footnote 1).

## Conflict of Interest Statement

Marco Gattorno and Alberto Martini has received honoraria for meeting presentations from Novartis and SOBI. The Gaslini hospital to which Alberto Martini and Marco Gattorno work as full-time employees have received contributions to support PRINTO and Eurofever research activities from Bristol-Myers Squibb, Abbott, Novartis, Roche, Centocor, ACRAF, Pfizer, and Xoma.

## Footnotes

^1^http://clinicaltrials.gov

^2^http://www.printo.it/eurofever/

^3^http://fmf.igh.cnrs.fr
